# Pan-Cancer Analysis of Head-to-Head Gene Pairs in Terms of Transcriptional Activity, Co-expression and Regulation

**DOI:** 10.3389/fgene.2020.560997

**Published:** 2021-01-07

**Authors:** Yunqin Chen, Hong Li, Yuan-Yuan Li, Yixue Li

**Affiliations:** ^1^School of Life Sciences and Biotechnology, Shanghai Jiao Tong University, Shanghai, China; ^2^CAS Key Laboratory of Computational Biology, CAS-MPG Partner Institute for Computational Biology, Shanghai Institute of Nutrition and Health, Shanghai Institutes for Biological Sciences, University of Chinese Academy of Sciences, Chinese Academy of Sciences, Shanghai, China; ^3^Shanghai Center for Bioinformation Technology, Shanghai, China; ^4^Shanghai Engineering Research Center of Pharmaceutical Translation, Shanghai, China

**Keywords:** head-to-head organization, housekeeping genes, differential co-expression, dysfunctional regulation, carcinogenesis

## Abstract

**Background:**

Head-to-Head (H2H) gene pairs are regulated by bidirectional promoters and divergently transcribed from opposite DNA strands with transcription start sites (TSSs) separated within 1 kb. H2H organization is ancient and conserved, and H2H pairs tend to exhibit similar expression patterns. Although some H2H genes have been reported to be associated with disease and cancer, there is a lack of systematic studies on H2H organization in the scenario of cancer development.

**Methods:**

Human H2H gene pairs were identified based on GENCODE hg19 and the functional relevance of H2H pairs was explored through function enrichment and semantic similarity analysis. To investigate the association between H2H organization and carcinogenesis, pan-cancer differential analysis of H2H genes about transcriptional activity, co-expression and transcriptional regulation by transcription factors and enhancers were performed based on data from The Cancer Genome Atlas. Cox proportional hazards regression model and log-rank test were used to determine the prognostic powers of H2H pairs.

**Results:**

In the present study, we first updated H2H genes from 1,447 to 3,150 pairs, from which the peak group with TSS distance of 1–100 was observed as expected in our previous work. It was found that housekeeping genes, mitochondrial-functional associated genes and cancer genes tend to be organized in H2H arrangement. Pan-cancer analysis indicates that H2H genes are transcriptionally active than random genes in both normal and cancer tissues, but H2H pairs display higher correlation in cancer than in normal. Particularly, housekeeping H2H pairs are differentially correlated much more significantly than non-housekeeping H2H pairs are. Some of differentially correlated H2H pairs were found to be associated with prognosis. The alteration of TF similarity seems to contribute to differential co-expression of H2H pairs during carcinogenesis; meanwhile remote enhancers also at least partly explain the differential co-expression and co-regulation of H2H pairs.

**Conclusion:**

H2H pairs tend to show much stronger positive expression correlation in cancer than in normal due to differential regulation of bidirectional promoters. The study provides insights into the significance of H2H organization in carcinogenesis and the underlying dysfunctional regulation mechanisms. Those differentially correlated H2H pairs associated with survival have the potential to be prognostic biomarkers and therapeutic targets for cancer.

## Introduction

Eukaryotic genomes are organized in a complex and hierarchical manner, and the regulation of gene expression is an intricate process that involves multiple levels of control. It has been well established that gene order in eukaryotic genomes is not completely random and genes clustered within the same genomic neighborhoods tend to have similar and/or coordinated expression (Hurst et al., 2004). Exploring the functional relevance and regulation mechanisms of neighboring genes is important for understanding eukaryotic chromosome organization and its association with disease (Hurst et al., 2004; Michalak, 2008).

**FIGURE 1:**
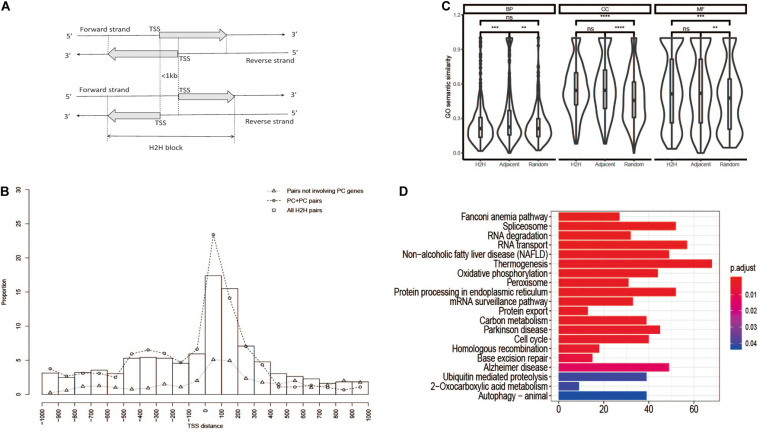
Characteristics of H2H organization. **(A)** Schematic diagram of H2H organization. **(B)** TSS distance distribution of H2H pairs. PC, protein coding. **(C)** GO semantic similarity of H2H pairs and random pairs. **(D)** Enriched signaling pathways of H2H genes.

It has been reported that more than 10% of human genes are divergently transcribed from opposite DNA strands with their transcription start sites (TSSs) separated within 1 kb (Adachi and Lieber, 2002; Trinklein et al., 2004; Li et al., 2006). This kind of gene arrangement is called head-to-head (H2H) (Figure 1A). The promoter region shared by two H2H genes is regarded as a bidirectional promoter. The transcriptional activation of H2H genes is much stronger than background according to active transcription associated chromatin signatures (Lin et al., 2007). Our previous work revealed that H2H gene organization was ancient and conserved during vertebrate evolution (Chen et al., 2010). The bidirectional arrangement seems to help genes be organized in an efficient way and contribute to the compactness of the overall gene regulatory network. In addition to expression correlation between two H2H genes, we also found that a gene pair involving two distinct H2H pairs tend to spatially interact with each other and be co-regulated by transcriptional factors like *CTCF*, *BDP1*, *GATA2*, and *POL3* (Chen et al., 2014).

Since a bidirectional promoter simultaneously regulate the transcription of two paired genes, genetic mutations or epigenetic changes in the promoter region could affect two involved genes and double the impact they might have in one single gene promoter. It was reported hyper-methylation of bidirectional promoter-associated CpG island was able to silence both genes simultaneously (Shu et al., 2006). In this sense, while achieving efficiency, H2H gene organization also put involved genes at greater risk. Sporadic studies have reported the association of H2H genes with DNA repair and carcinogenesis, such as a significant enrichment of bidirectional promoters for somatic breast cancer genes including *BRCA1*, *BRCA2*, *FANCB2*, and *FANCD* (Yang et al., 2007), and stronger selective pressures of methylation and copy number alteration on H2H organization in tumor samples (Thompson et al., 2018).

However, there are very few studies on the functional relevance and regulation mechanism of H2H genes in the scenario of tumor development. The rapid accumulation of genetic and transcriptomic data from both normal and tumor samples in public domain, such as the Cancer Genome Atlas (TCGA) (12), has provided sufficient opportunities for research in this area. In the work, we first updated H2H gene pairs and investigated the association of H2H gene arrangement with housekeeping genes, mitochondrial function-associated genes and cancer genes. Pan-cancer analysis indicated that H2H pairs exhibited much higher expression correlation in cancer than in normal, among which housekeeping H2H pairs are more likely to be differentially correlated between in cancer and normal state. Some of differentially correlated H2H pairs, or shifted pairs, were proposed to be novel prognostic biomarkers. We also explored the underlying mechanisms of differential transcriptional regulation of H2H pairs by transcriptional factors and enhancers. This study provides insights into the significance of this ancient gene organization, particularly in its role in cancer development and the potential to be predictive biomarkers and therapeutic targets for cancer.

## Materials and Methods

### Identification of Human Head-to-Head Gene Pairs

“Head-to-head” (H2H) is used to describe a gene arrangement where two neighboring genes are located on opposite DNA strands and transcribed divergently with TSSs less than 1 kb (see Figure 1A). The mitochondrial genome was ignored in this work because its gene organization is more compact than that of the nuclear genome. Gene pairs in which a gene was entirely located in another gene were excluded from our study. GENCODE hg19 gene annotation file^1^ was adopted to identify H2H gene pairs. The putative bidirectional promoter was defined as the region between two TSSs of an H2H pair.

Adjacent gene pairs refer to gene pairs that are linear neighbors in chromosomes with the same transcription direction, while random gene pairs refer to gene pairs that are randomly located and separated by other genes. Adjacent gene pairs and random gene pairs are taken as controls when investigating the characteristics of H2H pairs.

### Gene Expression Profile and Clinical Information From TCGA

By analyzing TCGA data including 20,531 genes’ expression profile in 11,069 patient cases across 33 tumor types and clinical data, the PanCanAtlas provides comprehensive genomic information to answer questions about cancer^2^.

### Housekeeping Genes, Mitochondrial Function-Associated Genes and Cancer Gene Consensus

Housekeeping genes are genes expressed in all tissues of an organism under normal conditions, and are required for the maintenance of basal cellular functions. A total of 3,804 housekeeping genes identified from expression profiling across16 normal human tissue types were available at http://www.tau.ac.il/elieis/HKG/ (Eisenberg and Levanon, 2013). A total of 1,455 mitochondrial function-associated genes were obtained from multiple sources, following a previously reported method (Billingsley et al., 2019). A total 723 genes containing mutations that have been causally implicated in cancer were obtained from Cancer genes COSMIC Cancer Gene Consensus (CGC) (Sondka et al., 2018).

### Function Enrichment and Semantic Similarity of H2H Pairs

R package “clusterProfiler” (Wang et al., 2012) was applied to perform functional enrichment analysis. Adjusted *p*-values less than 0.01 obtained by the BH method were regarded as statistically significant.

For each gene pair, we implemented “geneSim” function from R package “GOSemSim” (Yu et al., 2010) to calculate semantic similarity between two gene products by setting “measure” as “Wang” and “combine” as “BMA.” The Wang method (Wang et al., 2007) estimates the semantic similarity of two GO terms according to both the locations of these terms in the GO graph and their relations with their ancestor terms.

### Transcriptional Activity, Expression Similarity and TF Similarity

Transcriptional activity was used to evaluate the gene expression activity. We firstly ranked all gene expression values from low to high in each sample, and then the median rank of a gene across all samples was taken to score the gene’s transcriptional activity in normal or cancer state. The gene activity score was eventually scaled to 0–1 by dividing total gene number. The higher the score, the stronger the gene activity.

Expression similarity was represented by expression correlation, which was evaluated by Pearson correlation coefficient.

Transcription factor (TF) similarity of two genes was estimated by calculating the ratio of intersected TFs (common TFs) in all union TFs of the two H2H genes. Spearman correlation coefficients were used to evaluate the association of TF similarity and expression correlation of two genes in one H2H pair.

### Identification of Shifted H2H Pairs

“Shifted H2H pairs” were defined as lowly co-expressed H2H pairs in normal but highly correlated in cancer state. Chi-squared test was applied to test whether housekeeping genes were enriched in the shifted H2H pairs. We compared the proportions of pairs with 0-HK (no housekeeping gene), 1-HK (one housekeeping gene), and 2-HK (two housekeeping genes) between shifted H2H pairs and all H2H pairs. *P* < 0.05 was considered as statistically significant.

### Putative Enhancer-Gene Regulation and Transcription Factor Targeted Genes

The landscape of enhancer activities from the TCGA RNA-seq data was obtained from Chen et al. (2018), which is based on the high quality expressed enhancer annotations in FANTOM project (Andersson et al., 2014)^3^. Putative enhancer RNA (eRNA) target H2H genes were defined as eRNA-gene pairs with close distance (≤1 MB away from H2H block) and Spearman’s correlation coefficients ≥0.3 and FDR < 0.05 (Zhang et al., 2019). R package “GenomicRanges” was used to identify intersecting genomic regions (Lawrence et al., 2013). If both genes in a H2H pair regulated by a same enhancer, the H2H pair was regarded as enhancer-regulated H2H pair. To characterize the functional roles of eRNAs in cancer, we identified the differentially expressed eRNAs in cancer compared to in normal using the following criteria: expressed in at least three tumor-normal paired samples; absolute log2 (fold change) >log (1.5) and BH-adjusted *p* value <0.05 by paired *t* test.

Transcription factors were collected from literature of Lambert et al. (2018). We identified putative regulators of genes based on the correlation between individual gene and each TF in a given cancer type, and considered Spearman’s correlation coefficients ≥0.3 and FDR <0.05 as significant.

### Integrative Analysis of Expression Score of H2H Pairs With Clinical Data

In each sample, the expression levels of two genes in a pair underwent pairwise comparison to generate a score to represent the pair’s status. If the first gene of one gene pair had a higher expression level than the second one, a gene pair score of 1 was assigned; otherwise, the gene pair score was 0. R package “survival” was applied to perform the integrative analysis of expression score of H2H pairs with clinical data (Therneau and Lumley, 2019). For each H2H pair, we used cox proportional hazards regression model and log-rank test to determine its prognostic power. H2H pairs associated with either the overall survival time or disease-free time (*p* value < 0.05) were considered as prognostic pairs.

### All Statistical Analyses

All statistical analyses were done by using software R^4^. Pearson correlation coefficient was used to evaluate the gene pair’s expression correlation. Spearman correlation coefficient was used to evaluate the association of transcriptional regulators with genes. All pooled samples in TCGA contained 10,332 cancer samples and 737 normal samples from 33 tumor types. For the comparison between cancer and normal, only 15 tumor types with more than 10 normal samples were selected for subtype analysis: BLCA, bladder urothelial carcinoma; BRCA, breast invasive carcinoma; COAD, colon adenocarcinoma; ESCA, esophageal carcinoma; HNSC, head and neck squamous cell carcinoma; KICH, kidney chromophobe; KIRC, kidney renal clear cell carcinoma; KIRP, kidney renal papillary cell carcinoma; LIHC, liver hepatocellular carcinoma; LUAD, lung adenocarcinoma; LUSC, lung squamous cell carcinoma; PRAD, prostate adenocarcinoma; STAD, stomach adenocarcinoma; THCA, thyroid carcinoma; UCEC, uterine corpus endometrial carcinoma.

## Results

### Identification and Characterization of Human Head-to-Head Gene Pairs

According to the definition of H2H gene organization (Figure 1A), we identified 1,262 human H2H pairs from 26,813 human genes according to the genomic mapping data from NCBI BUILD.35.1 in 2006 (Shu et al., 2006), which was updated to be 1,447 pairs from 28,924 genes according to NCBI_Build_36.2 in 2009 (Yu et al., 2009). In the current work, we identified 3,150 human H2H pairs from 57,800 genes based on GENCODE hg19. The 3,150 pairs involved a total of 6,283 genes, indicating that about 11% human genes are organized in H2H configuration. Although the H2H pair number has increased dramatically due to the significant accumulation of gene annotation information, the proportion is only a little higher than our previous reports, 9.4% in 2006 (Shu et al., 2006) and 9.8% in 2009 (Yu et al., 2009).

Among the 3,150 pairs, a total of 2,740 (87%) H2H pairs involved at least one protein coding gene, including 1,197 (38%) “protein coding – protein coding” type, 935 (30%) “protein coding – antisense” type, 377 (12%) “protein coding – lncRNA” type, 129 (4%) “protein coding – pseudogene” type, and 25 (1%) “protein coding – miRNA” type (see Supplementary Table 1 for more types), suggesting some non-coding RNA transcriptions could couple the expression of protein-coding genes through local effects of bidirectional promoters (Wei et al., 2011; Scruggs et al., 2015).

We characterized the structural features of head-to-head gene organization using the distribution of TSS distance (Figure 1B). Similar to our previous observation (Shu et al., 2006; Chen et al., 2010), the pairs with TSS distance of 1–400 account for a large proportion (1,409, 44.7%) of all H2H pairs, and those protein coding H2H pairs (PC-PC) with TSS distance of 1–400 represent an even larger proportion (585, 48.9%) of PC-PC H2H pairs. It was noted that the peak is the region of [1, 100], which validated our previous guess that “an impending replacement of the peak column [100, 200] by [0, 100] in future data updates” (Chen et al., 2010). Considering that the core promoter is defined as the DNA segment of 1–100 bp region upstream of a TSS (Roeder, 1996), bidirectional promoters falling within the core promoter region are most likely to efficiently co-regulate the transcription of two divergent genes.

### Functional Relevance and Importance of Head-to-Head Gene Pairs

When both genes of a H2H pair could be annotated by any terms from Gene Ontology, the pair was denoted as an “annotated pair.” Out of the 3,150 H2H pairs, 703, 791, and 801 annotated pairs were obtained corresponding to the three subsystems, “Biological Process” (BP), “Molecular Function” (MF), and “Cellular Component” (CC), respectively. Graph-based Wang method (Wang et al., 2007) was used to evaluate semantic similarity of GO terms. As shown in Figure 1C, both H2H pairs and adjacent pairs showed significantly higher functional similarity in terms of molecular function and cellular component than random pairs. But for the “biological process” (BP) which represents biological objectives, no significant difference of functional similarity was observed between H2H pairs and random pairs, suggesting the selection of H2H organization may not be based on functional relevance.

KEGG pathway enrichment analysis showed that H2H genes were primarily involved in some fundamental functions such as “Fanconi anemia pathway,” “Spliceosome,” “RNA degradation,” “RNA transport,” “Oxidative phosphorylation,” “Cell Cycle,” “mRNA surveillance pathway,” and so on (Figure 1D). It was similar with previous studies (Trinklein et al., 2004; Li et al., 2006; Xu et al., 2012).

In the very first genome-scale analysis of H2H gene pairs in 2002 (3), it was pointed out that 30% of 270 housekeeping (HK) genes of the time were regulated by bidirectional promoters. In this work, we re-checked the association of housekeeping genes with H2H organization based on an updated housekeeping gene lists (12) (see section “Materials and Methods”) and found that among the 3,589 housekeeping genes, 1,270 were involved in H2H pairs, accounting for 35% of housekeeping genes (*p*-value < 0.001). The proportion, or the tendency, is basically consistent with the observation reported in 2002. The 3,150 H2H pairs were then categorized into three subgroups according to the number of involved housekeeping genes, consisting of 2,067 H2H pairs without any housekeeping genes, 896 pairs with one housekeeping gene, and 187 pairs with two housekeeping genes. That is, a total of 1,083 (34%) H2H pairs involve at least one housekeeping gene. It is interesting that the proportion of H2H pairs of the three subgroups in all adjacent gene pairs displayed a gradually incremental trend, which is 6% (no HK genes), 15% (one HK gene), and 27% (two HK genes), with *p*-value less than 0.001 by chi-squared test. We have reported in our previous work that there is a negative selection on the separation of H2H gene pairs during vertebrate evolution (Shu et al., 2006); herein, housekeeping functions have been exerting extra selective pressure on H2H gene organization. In other words, divergent bidirectional transcription seems to benefit for maintaining the fundamental functions of housekeeping genes.

It is noticeable that the enrichment analysis based on Gene Ontology (Supplementary Figure 1) indicated that H2H genes were overrepresented in mitochondrial function such as “mitochondrial organization” (BP), “mitochondrial inner membrane” (CC), and “NADH dehydrogenase activity” (MF). According to mitochondrial function related gene lists from multiple sources (see section “Materials and Methods”), 390 out of 1,455 mitochondrial function-associated genes were organized in a head-to-head manner with a higher proportion (27%) than random genes (11%), with *p* value less than 0.001 by chi-square test. Besides the functional importance of mitochondrial genes, the endosymbiotic theory of mitochondrial evolution helps to explain the enrichment of H2H gene organization in mitochondrial genes (Chang et al., 2010; Zimorski et al., 2014). That is, since it is believed that mitochondria originated from prokaryotic cells and most mitochondrial genes have been horizontally transferred to host genome, mitochondrial function associated genes are more likely to keep compact organization than the other eukaryotic genes.

Considering the observation that H2H genes are significantly involved in some fundamental functions, such as DNA repair systems and respiratory complex of the mitochondrial electron transport chain, we proposed that H2H gene organization might be associated with carcinogenesis (Uchiumi et al., 2015; Urra et al., 2017; Sica et al., 2019) although there are very few of studies on this topic (Yang et al., 2007; Thompson et al., 2018). We found that 23% cancer genes from COSMIC Cancer Gene Consensus (CGC) (Sondka et al., 2018) were involved in H2H gene organization, significantly higher than random genes (11%) with p less than 0.001 by chi-square test.

Among the three groups of genes, housekeeping genes, mitochondrial genes and cancer genes, according to the proportion of genes involved in H2H pairs, 35, 27, and 23%, we propose that the separation of housekeeping H2H pairs is under the strongest negative selection pressure.

### High Transcriptional Activity of H2H Genes

Given that bidirectional promoters of H2H pairs were enriched with hypersensitive DNase and modified histones associated with transactional activation (Lin et al., 2007; Chen et al., 2014), H2H genes seem to be in an active state. Considering the association of H2H gene organization with housekeeping functions and carcinogenesis, we set out to systematically investigate the transcriptional activity of H2H genes in both normal and cancer state based on TCGA datasets. A total of 3,605 H2H genes have expression profiles from 15 types of tumor (see section “Materials and Methods” for cancer type selection). We first ranked all gene expression values from low to high in each sample, and then the median rank of a gene across all samples was taken to score the gene’s transcriptional activity. The gene activity score was eventually scaled to 0–1 by dividing total gene number. The higher the score, the stronger the gene activity.

**FIGURE 2:**
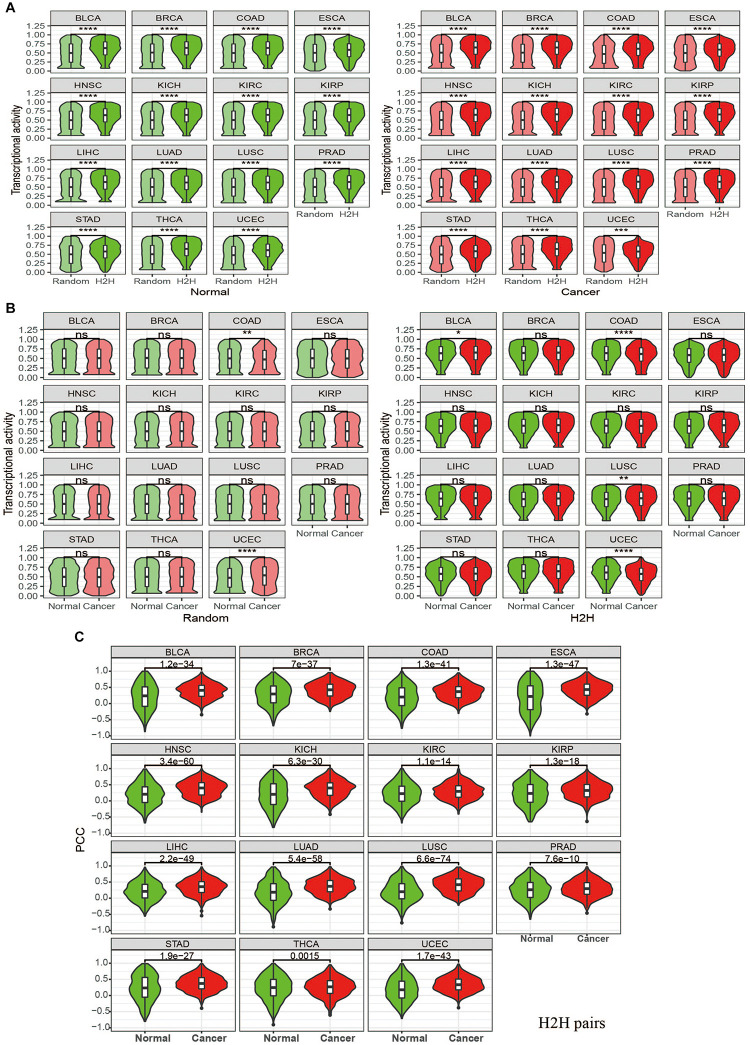
Pan-cancer analysis of transcriptional activity of H2H genes and expression correlation of H2H pairs in tumor and normal state using TCGA datasets. **(A)** Comparison of transcriptional activity between H2H genes and random genes among 15 tumor types when in both normal or cancer state. **(B)** Paired comparison of transcriptional activity between in cancer and normal state in 15 tumor types for H2H genes and random genes, respectively. **(C)** Comparison of expression correlations for H2H pairs between in cancer and normal state in 15 tumor type. Random genes were marked with light colors (“light green” for normal state and “light coral” for cancer state) while H2H genes were marked with dark colors (“green” for normal state and “red” for cancer state). “ns” means *p* value >0.05, not significant; “*” means *p* value <0.05; “**” means *p* value <0.01; “***” means *p* value <0.001 and “****” means *p* values < 0.0001. BLCA, bladder urothelial carcinoma; BRCA, breast invasive carcinoma; COAD, colon adenocarcinoma, ESCA, esophageal carcinoma; HNSC, head and neck squamous cell carcinoma; KICH, kidney chromophobe; KIRC, kidney renal clear cell carcinoma; KIRP, kidney renal papillary cell carcinoma; LIHC, liver hepatocellular carcinoma; LUAD, lung adenocarcinoma; LUSC, lung squamous cell carcinoma; PRAD, prostate adenocarcinoma; STAD, stomach adenocarcinoma; THCA, thyroid carcinoma; UCEC, uterine corpus endometrial carcinoma.

**FIGURE 3:**
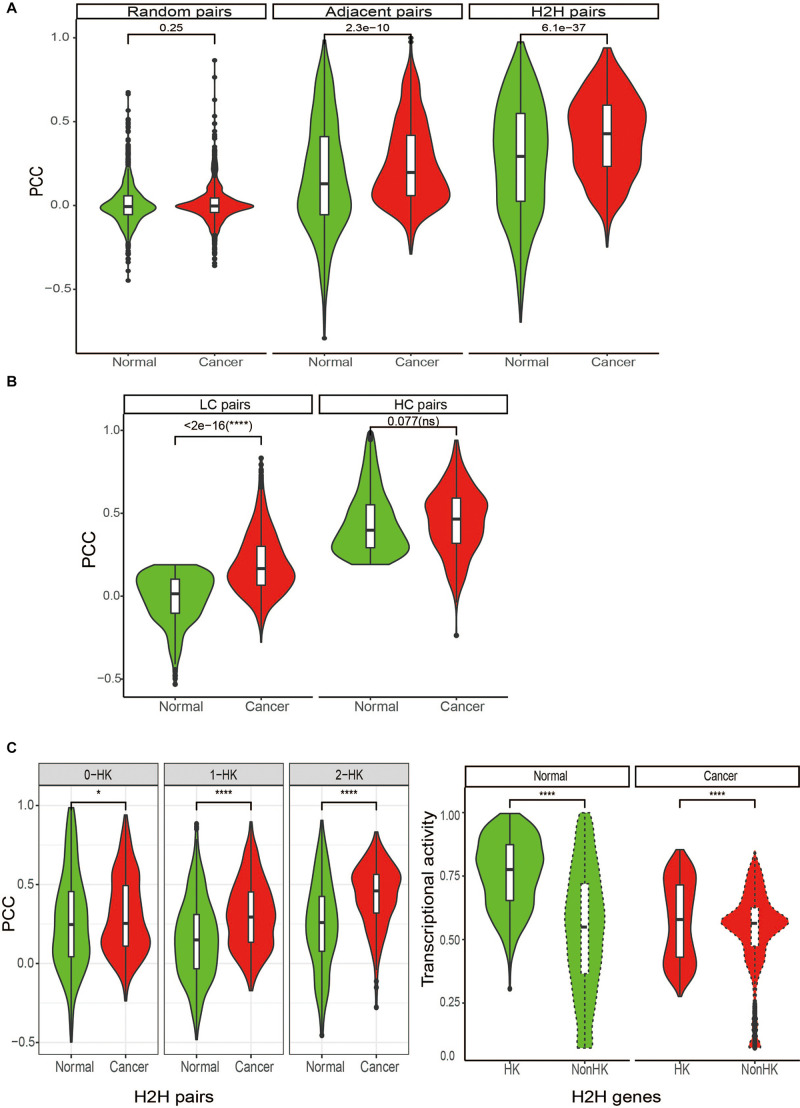
Differential expression correlation of H2H pairs between in tumor and normal state. **(A)** Comparison of expression correlations for H2H pairs, adjacent pairs and random pairs between in cancer and normal stateregardless of tumor types. **(B)** Comparison of expression correlations between in cancer and normal state for two subgroups based on the median value of expression correlation in normal (0.19): LC pairs and HC pairs. LC: lowly correlated pairs in normal with PCC < 0.19; HC: low correlated pairs in normal with PCC ≥ 0.19. PCC: Pearson correlation coefficients. **(C)** Comparison of expression correlations of H2H pairs among three groups based on the number of housekeeping genes in a pair and transcriptional activities of housekeeping H2H genes. HK, housekeeping; 0-HK, no housekeeping genes involved in a H2H pair; 1-HK, one housekeeping gene involved in a H2H pair; 2- both housekeeping genes forming a H2H pairs.

It was found that the 3,605 H2H genes with bidirectional promoters display much higher activity than 3,605 randomly selected genes with unidirectional promoters across each tumor type, no matter in cancer or in normal state (Figure 2A); meanwhile, the gene activity does not show any significant difference between cancer and normal among nearly all cancer types, no matter for H2H genes, or for random genes (Figure 2B). The same phenomena were observed in pan-cancer samples regardless of tumor type. As a significantly high proportion of housekeeping genes are organized in head-to-head manner, we paid special attention to those housekeeping H2H genes. Among the 3,605 H2H genes with expression data available, 1,190 are housekeeping genes and 2,415 are not housekeeping genes. It is interesting that housekeeping H2H genes are more active than non-housekeeping H2H genes both in cancer and normal state (Figure 3C).

### Coherent Expression Correlation of H2H Pairs and Their Differential Co-expression in Cancer

We considered Pearson correlation coefficient (PCC) of expression profiles of two genes in a pair’ to evaluate the gene pair’s expression similarity. First of all, there were a total of 964 H2H pairs with TCGA expression data available for both genes. In order to compare the Pearson correlation coefficient (PCC) of H2H pairs with that of adjacent pairs and random pairs, we randomly selected 964 adjacent pairs and 964 random pairs from the identities in the expression profiles. Pearson correlation coefficients were calculated between two genes involved in every gene pairs in cancer or in normal. As expected, H2H pairs showed the highest expression correlation, followed by adjacent pairs, both in normal and cancer states; in comparison, random pairs didn’t show any correlation (Figure 3A). Although H2H pairs showed both high expression activity and coherent expression correlation, no significant relationships between expression activity and expression correlation was observed for H2H pairs.

Since differential correlation analysis is useful for deciphering dysfunctional regulations or decoupled interactions during phenotypic changes (de la Fuente, 2010; Yang et al., 2013), we then compared the expression correlation of H2H pairs between in cancer and normal and checked the differential co-expression by measuring the difference of Pearson correlation coefficients, i.e., PCC (cancer)-PCC(normal). Pan-cancer analysis showed that H2H pairs had significantly stronger positive correlation in cancer than in normal (Figure 3A regardless of tumor types and Figure 2C for each tumor type). The average and median differential co-expression was 0.11 and 0.1 in pooled samples, which was significantly different from the H0 hypothesis (paired Wilcoxon test, *p* < 0.001). We categorized the 964 H2H pairs into three subgroups according to the number of housekeeping genes involved in one H2H pair, and obtained 402 (41.7%) pairs without any housekeeping genes, 401 (41.6%) pairs with one housekeeping gene, and 162 (16.8%) pairs with two housekeeping genes. Similar to the above trend hold by all 964 H2H pairs, each subgroup displayed significantly stronger positive correlation in cancer than in normal no matter in pooled samples; meanwhile, the subgroup with two housekeeping genes possess the highest PCC in cancer and the highest PCC difference between cancer and normal (Figure 3C). The phenomena were seen in each tumor type (data not shown). It was noticed that the differential co-expression increased gradually among the three group with the number of housekeeping gene involved in H2H pairs (Supplementary Figure 2), implying the relevance of housekeeping H2H genes in carcinogenesis in terms of differential co-expression and differential regulation.

We then divided the 964 H2H pairs into two subgroups based on the median value of expression correlation in pooled normal samples (0.19), and obtained “LC” pairs with PCC less than 0.19 and “HC” pairs with PCC not less than 0.19. Note that “LC” pairs could be weakly correlated and negatively correlated as well. It was found that “LC” H2H pairs tended to shift to be positively correlated in cancer with much larger change than “HC” H2H pairs did (Figure 3B). We therefore defined a special type of differentially correlated H2H pairs, “shifted H2H pairs” as those with PCC in normal less than the median value (0.19) and PCC in cancer not less than the median value (0.31), and obtained a total of 116 shifted H2H pairs out of the 964 pairs using pooled cancer and normal samples. It was found that 83% shifted pairs involved housekeeping genes with statistically significant association (Table 1, *p* < 0.001 by chi-squared test), and the proportion of H2H pairs with two HK genes jumped from 16.8% of the 964 H2H pairs to 31% of the 116 shifted pairs.

**TABLE 1:** Shifted H2H pairs were prone to involve in housekeeping genes compared to the background of all 964 expressed H2H pairs.

	Shifted H2H pairs	Background H2H pairs	*P* value
**HK H2H, *n*(%)**			<0.001
0-HK	20 (17.2%)	401 (41.6%)	
1-HK	60 (51.7%)	401 (41.6%)	
2-HK	36 (31.0%)	162 (16.8%)	

*Chi-squared test was used. 0-HK, two genes in a H2H pair are not housekeeping genes; 1-HK, one gene in a H2H pair belongs to housekeeping genes; 2-HK, both two genes in a H2H pairs are housekeeping genes.*

We noticed that the shifted H2H pairs primarily preferred “oxidative phosphorylation” pathway. Considering the dysregulation of “oxidative phosphorylation” pathway genes has been associated with malignancies, and even become potential targets for cancer therapy (Ashton et al., 2018), we therefore explored clinical relevance of these shifted H2H pairs by using survival analysis. The comparison of two genes’ expression levels in an H2H pair was taken as an indicator (see section “Materials and Methods”). Specifically, a pair with score 1 or 0 represents that the expression level of the first gene is higher or lower than that of the second gene. We finally identified 57 shifted H2H pairs which are individually associated with overall survival (OS) and disease-free survival (DFS) (see Supplementary Table 2). “*METTL4*-*NDC80*,” “*C1orf109*-*CDCA*,” and “*TMEM60*-*PHTF2*” with the score of 1 consistently played protective roles across multiple types of cancer, while pairs like “*ATAD2*-*WDYHV1*” and “*AURKA*-*CSTF1*” with the score of 1 seemed to be associated with cancer progression and a worse survival across multiple cancer types.

### Differential Regulation of Bidirectional Promoters in Cancer

**FIGURE 4:**
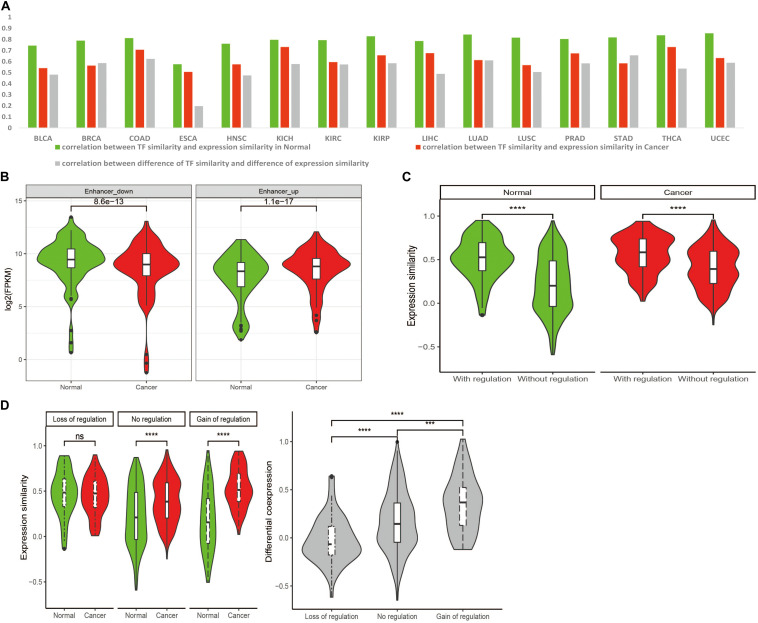
Transcriptional regulation of transcription factor (TF) and enhancer on expression and co-expression of H2H pairs. **(A)** Spearman correlation coefficients of TF similarity and expression similarity, TF similarity difference and differential co-expression in 15 tumor types for H2H pairs. **(B)** The differential expression of H2H genes regulated by differentially expressed eRNAs. “enhancer-up”: H2H genes targeted by up-regulated eRNAs in cancer than in normal; “enhancer-down”: H2H genes regulated by down-regulated eRNAs in cancer than in normal. **(C)** H2H pairs with enhancer regulation showed higher expression correlation than those without enhancer regulation. **(D)** Enhancer regulation affected differential co-expression for H2H pairs between in cancer and normal. “Loss of regulation”: H2H pairs which were regulated by enhancers only in normal and lost enhancer-regulation in cancer; “Gain of regulation”: H2H pairs which didn’t have enhancer regulation in normal but gained enhancer-regulation in cancer; “No regulation”: H2H pairs without enhancers regulation in both and normal states.

Our previous work (Chen et al., 2010) proposed that H2H pairs tend to be regulated by common TFs and thus achieve high expression correlation. The putative TFs of each H2H gene were identified for each cancer based on TF-target relationships and the expression profile (see section “Materials and Methods”). Then the TF similarity of two genes within each pair was estimated by calculating the ratio of shared TFs in union TFs of the two H2H genes. Spearman correlation coefficients were used to evaluate the association of TF similarity and expression correlation of two genes in one H2H pair. Across nearly all cancer types, the correlations between TF similarity and expression correlation in normal were stronger than that in cancer (Figure 4A), implying that the dominant role of TF regulation mechanism was weakened during carcinogenesis and other regulatory elements may be involved. We still observed a positive correlation between TF similarity difference and differential co-expression (Figure 4A), indicating the contribution of TF co-regulation to the positive correlation of H2H pairs during carcinogenesis (Figures 2C,  3A).

Since “LC” H2H pairs with low expression correlation in normal had a larger differential co-expression between cancer and normal than “HC” pairs did (Figure 3B), we checked the TF similarity difference of “LC” pairs between cancer and normal, and found that “LC” pairs had a low TF similarity in normal which shifted to be higher in cancer (Wilcoxon test, *p* < 0.001). This was consistent with the significant correlation between TF similarity difference and differential co-expression observed in the 3rd column in Figure 4A. That is, the more common TFs, the stronger co-regulation, and the higher positive correlation of H2H pairs.

Enhancers are *cis*-regulatory DNA elements associated with gene expression regulation (Schmitt et al., 2016). Through long-range chromosomal interactions, enhancers can spatially interact with their target promoters to regulate down-stream genes (Schoenfelder and Fraser, 2019). Whether enhancers play a role in expression of H2H paired genes is far from clear. Given the expression level of enhancer RNAs (eRNAs) represents an essential signature of enhancer activation (Murakawa et al., 2016), we adopted the eRNA expression profile identified from TCGA RNA-seq data to study the enhancer regulation during carcinogenesis (Chen et al., 2018; Zhang et al., 2019). Taking BRCA as an example, the putative enhancer regulated-H2H genes were identified based on location (≤1 MB away from H2H block) and expression correlation (see section “Materials and Methods”). A total of 827 differentially expressed eRNAs were first identified, then we investigated the expression of H2H genes regulated by those eRNAs in both normal and cancer states. A total of 112 H2H genes targeted by up-regulated eRNAs tended to have significantly higher expression levels in cancer compared with normal, while 69 H2H genes targeted by down-regulated eRNAs showed significantly lower expression levels in cancer than in normal (Figure 4B), confirming the role of enhancers in regulating H2H genes expression.

The H2H pair whose two genes were regulated by a same enhancer was regarded as an enhancer-regulated H2H pair. Among 452 H2H pairs with expression data of both genes available and candidate enhancers located within 1 MB distance, we identified 100 enhancer regulated-H2H pairs in normal state and 90 enhancer regulated-H2H pairs in cancer state. H2H pairs with enhancer regulation showed significantly higher expression correlation than the H2H pairs without enhancer regulation both in normal and in cancer states (see Figure 4C). Excluding 38 H2H pairs regulated by enhancers both in normal and cancer, there were 62 normal-specific H2H pairs which lost enhancer regulation in cancer (“loss of regulation” H2H) and 52 cancer-specific enhancer-regulated H2H pairs which gained enhancer regulation during carcinogenesis (“gain of regulation” H2H). It was noticed that the “gain of regulation” H2H pairs showed the highest differential co-expression between in cancer and normal, followed by the H2H pairs without enhancer regulation; while no significant differential co-expression was observed for “loss of regulation” H2H pairs between in cancer and normal (Figure 4D). It seemed that the significant differential correlation of H2H pairs could be enhanced by gain of enhancer regulation and compromised by loss of enhancer regulation. These results suggested the co-regulating function of enhancers imposing on H2H pairs and its potential role in carcinogenesis by its differential co-regulation of H2H pairs.

## Discussion

Since the first systematic study of H2H gene organization in 2002 (Adachi and Lieber, 2002), this special pattern of genome configuration has attracted attention for almost two decades. In the work, we updated H2H pairs by using hg19, and found that about 11% human genes are organized in the head-to-head arrangement. The peak group of H2H pairs with TSS distance of 1–100 was observed as we expected 10 years ago (Figure 1B). It was found that housekeeping genes, mitochondrial-functional associated genes and cancer genes tend to be organized in H2H arrangement. H2H genes are transcriptionally active than random genes in both normal and cancer tissues, and housekeeping H2H genes are more active than non-housekeeping H2H genes (Figure 2). The transcription of H2H pairs are much more correlated in cancer than in normal (Figure 2C). It is noticeable that housekeeping H2H pairs are more likely to be differentially correlated between in cancer and normal state (Figure 3C and Table 1). Considering the fundamental cellular function of housekeeping genes, it is meaningful to study H2H organization and its association with cancer development.

TFs shared by bidirectional promoters play a key role in co-regulating H2H gene pairs and here we propose that the alteration of TF similarity contributes to differential expression correlation during carcinogenesis. More common TFs lead to stronger co-regulation, and thus higher positive correlation between two genes involved in an H2H pair. Pan-cancer analysis showed that nearly across all 15 cancer types, the correlations between the expression similarity and TF similarity in normal were stronger than that in cancer (Figure 4A), suggesting that the contribution of TFs to transcriptional regulation varies from normal to cancer (Krasnov et al., 2016). Abnormal transcriptional mechanisms like aberrant methylation, mutations in promoter, chromatin remodeling, may participate in regulating bidirectional gene expression and weaken the dominant effect of TF co-regulation. We found that enhancers also participate in the transcriptional regulation of H2H genes (Figure 4B) and may play a potential role in carcinogenesis by differentially co-regulating H2H pairs (Figures 4C,D). A group of significantly differentially correlated H2H pairs, or shifted pairs, proved to enrich housekeeping genes, and some of the shifted pairs were proposed to be novel prognostic biomarkers, indicating the association of differential co-expression of H2H pairs with carcinogenesis (Supplementary Table 2).

There are several H2H pairs which have been reported as potential targets in the diagnosis and treatment of different cancer types. H2H pair PRR11-SKA2, sharing a NF-Y-regulated bidirectional promoter, is essential for the accelerated proliferation and motility of lung cancer cells and knockdown of the gene pair remarkably reduced cell proliferation, migration, and invasion in lung cancer cells (Wang et al., 2015). In another study, co-regulation and functional co-operativity of a H2H pair, *FOXM1* and *RHNO1*, was also proved in ovarian cancer. FOXM1-RHNO1 cooperatively promoted high-grade serous ovarian cancer (HGSC) cell growth, and knockdown of the two genes sensitized HGSC cells to the PARPi olaparib and mitigates acquired olaparib resistance (Barger et al., 2019). Here is a very recent report that PLAGL2-POFUT1 were proved to synergistically promote colorectal tumorigenesis by maintaining stemness of colorectal cancer stem cells, and modifying or editing transcriptional binding sites in their bidirectional promoter simultaneously suppressed both *PLAGL2* and *POFUT1* expression, which could offer promising therapeutic approaches (Li et al., 2019). Combined with these individual experimental evidences, we believe that our systematic observations provide valuable resources and clues for identifying predictive biomarkers and drug targets for cancer.

As the first systematic study of transcriptional activity and differential co-expression of H2H pairs in the scenario of cancer, we provide insights into the significance of H2H organization in carcinogenesis and the underlying dysfunctional regulation mechanisms. Considering that genetic or epigenetic alterations of bidirectional promoter regions could cause the functional consequences of both genes in a pair, we believe that integrating epigenetic information including histone modifications, methylation and 3D-chromatin structures to the present analysis pipeline would help to investigate the hidden complexity of the regulation mechanisms of bidirectional promoters during cancer development.

## Data Availability Statement

The original contributions presented in the study are included in the article/Supplementary Material, further inquiries can be directed to the corresponding author/s.

## Author Contributions

YL, Y-YL, and YC conceived this study. YC analyzed the data. HL provided suggestions about study design. All authors participated in writing the manuscript. All authors have read and approved the final manuscript.

## Conflict of Interest

The authors declare that the research was conducted in the absence of any commercial or financial relationships that could be construed as a potential conflict of interest.

## Footnotes

1 https://www.gencodegenes.org/releases/19.html

2 https://gdc.cancer.gov/about-data/publications/pancanatlas

3 http://fantom.gsc.riken.jp/5/datafiles/latest/extra/Enhancers/human_permissive_enhancers_phase_1_and_2.bed.gz

4 https://www.r-project.org/

## Supplementary Material

Supplementary Figure 1 Enriched GO terms of H2H genes in three subsystems: biological process (BP), molecular function (MF), cellular component (CC).

Supplementary Figure 2 Differential co-expression increased gradually among the three group with the number of housekeeping gene involved in H2H pairs.

Supplementary Table 1 Frequency of biotypes of H2H gene pairs.

Supplementary Table 2 Shifted H2H gene pairs associated with overall survival (OS) and disease free survival (DFS).
