# Supplementary figures and images for: Pan-Cancer Analysis of Head-to-Head Gene Pairs in Terms of Transcriptional Activity, Co-expression and Regulation
Source: Front Genet. 2021 Jan 7;11:560997. doi: 10.3389/fgene.2020.560997 (PMC7817982; doi:10.3389/fgene.2020.560997)

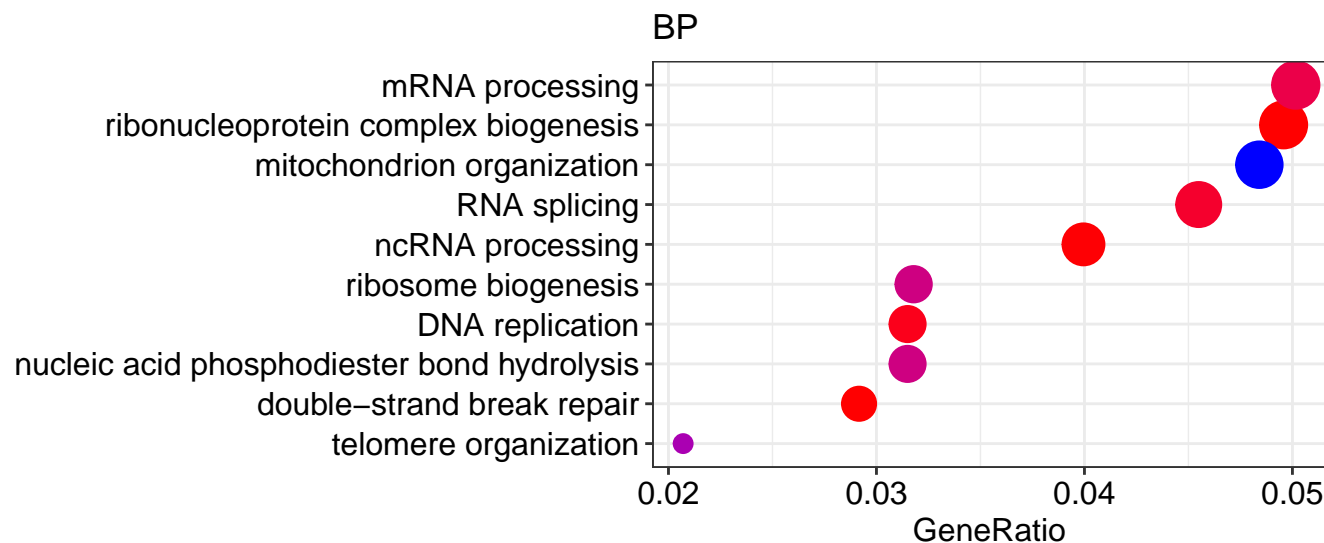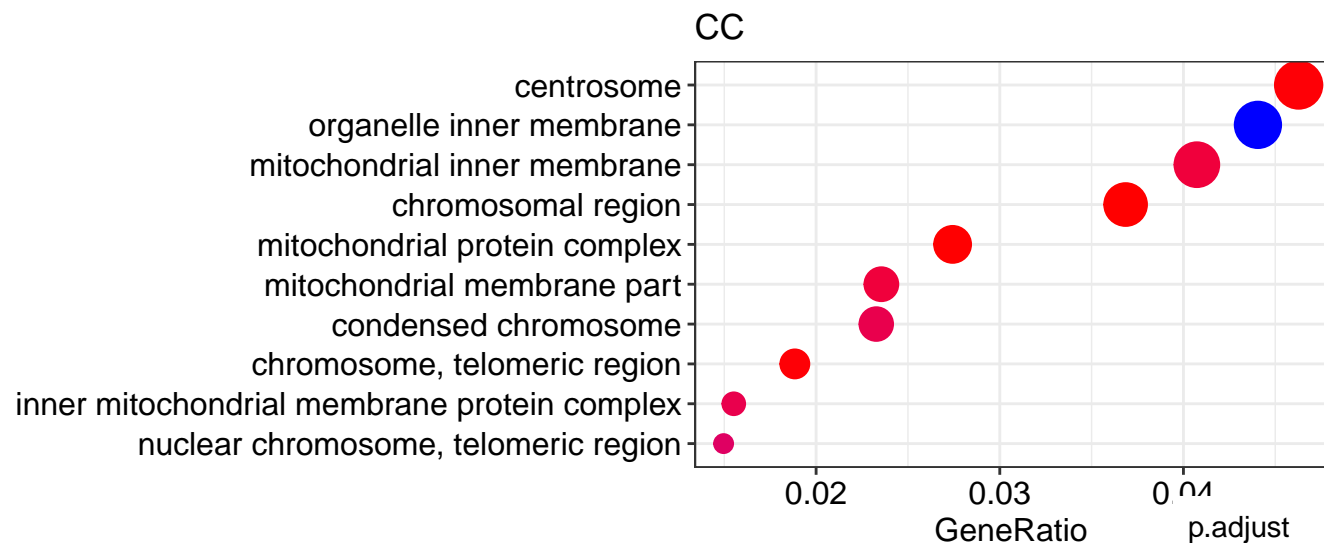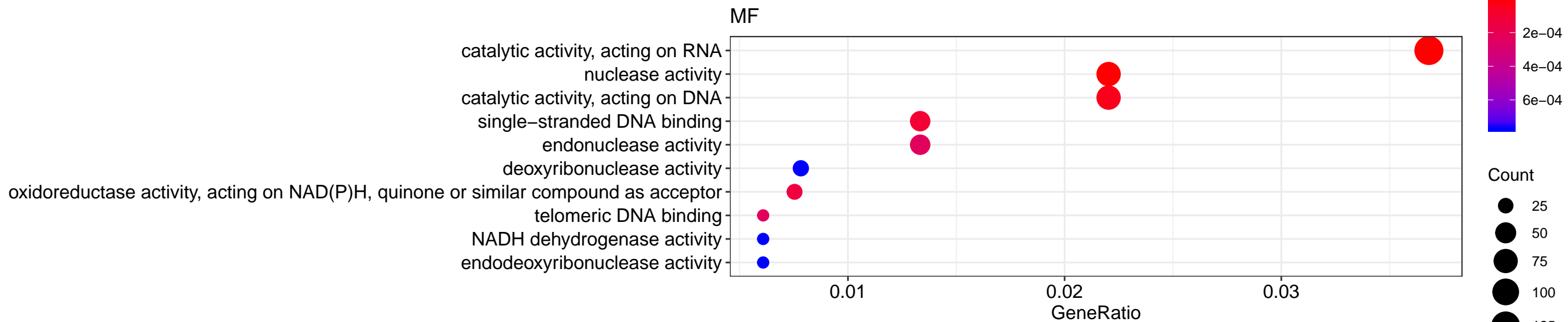

Supplement: Supplementary Figure 1 — Enriched GO terms of H2H genes in three subsystems: biological process (BP), molecular function (MF), cellular component (CC). [file Image_1.PDF]

Differential correlation

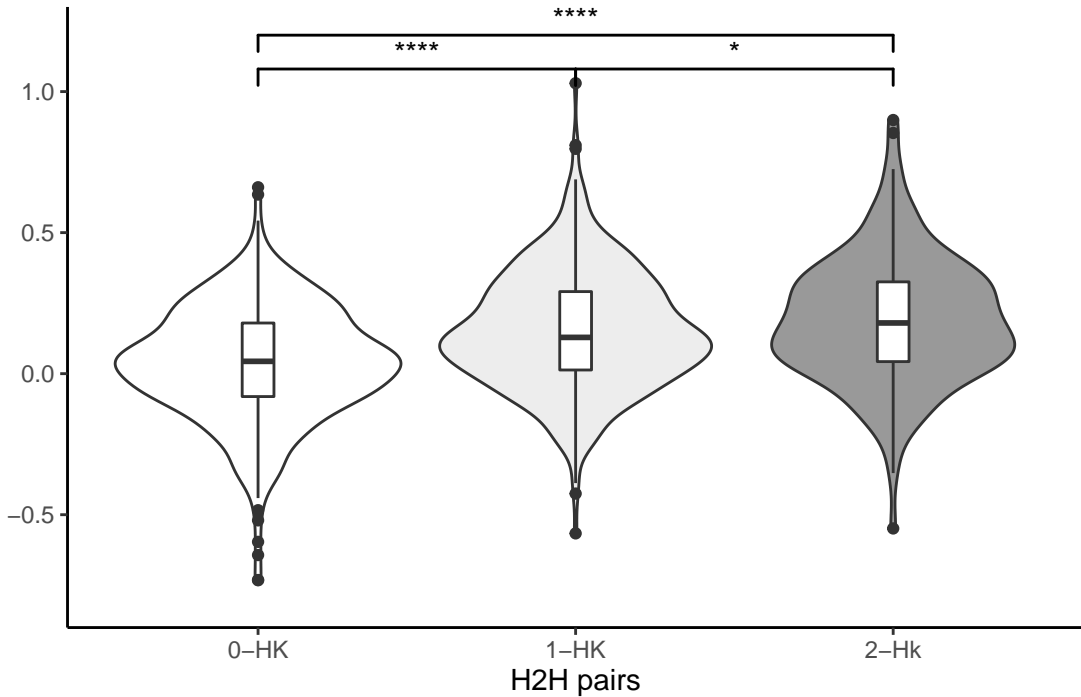

Supplement: Supplementary Figure 2 — Differential co-expression increased gradually among the three group with the number of housekeeping gene involved in H2H pairs. [file Image_2.PDF]
